# Environmental manipulation and stimulation of atypical children trough art therapy

**DOI:** 10.1192/j.eurpsy.2023.1738

**Published:** 2023-07-19

**Authors:** L.-M. Hosu

**Affiliations:** Community Social Services Complex for Children and Adult, GENERAL DIRECTORATE OF SOCIAL ASSISTANCE AND CHILD PROTECTION, COUNTY COMMUNITY CENTER CLUJ COUNTY COUNCIL, CLUJ-NAPOCA, Romania

## Abstract

**Introduction:**

The accumulated experience, both with typical and atypical children, led to the desire for an easier integration into the collective.Detecting and encouraging the skills of the atypical child, with the help of combined arts techniques and environmental stimulation, supports their’s integration into the comunity.

**Objectives:**

Beneficiaries-Centered Art therapy, with the aim of identifying and developing outstanding skills, trough environmental manipulation and combined arts techniques

**Methods:**

The environmental manipulation method is used in the art therapy session trough artistic installations and colorful fabrics. Trough chromatics and textures are generated different contexts and atmospheres sense, through which the atypical child comes to accept and discover that environment. Through the manipulation of artistic installations, gross motor skills are also required, having to produce large movements with the whole body, in order to shape the elements in the environment.

Costume making as a transitional phase in the adaptation of the beneficiary to the previously created environment independently or together with the art therapist, consists in harmonizing the beneficiary with the environment. These stages are achieved through mixed techniques of artistic work, painting, decorating, modeling, collage, weaving and binding techniques.

**Results:**

By practicing these methods during the art therapy sessions, the beneficiaries increased their self-esteem through the achievements they had and discovered and improved their outstanding skills: fine and gross motor skills, color sense, visual thinking, hand-eye coordination; improved and developed both verbal and non-verbal communication between beneficiary - art therapist and beneficiary - beneficiary, in the case of group sessions

**Image:**

**Figure:**
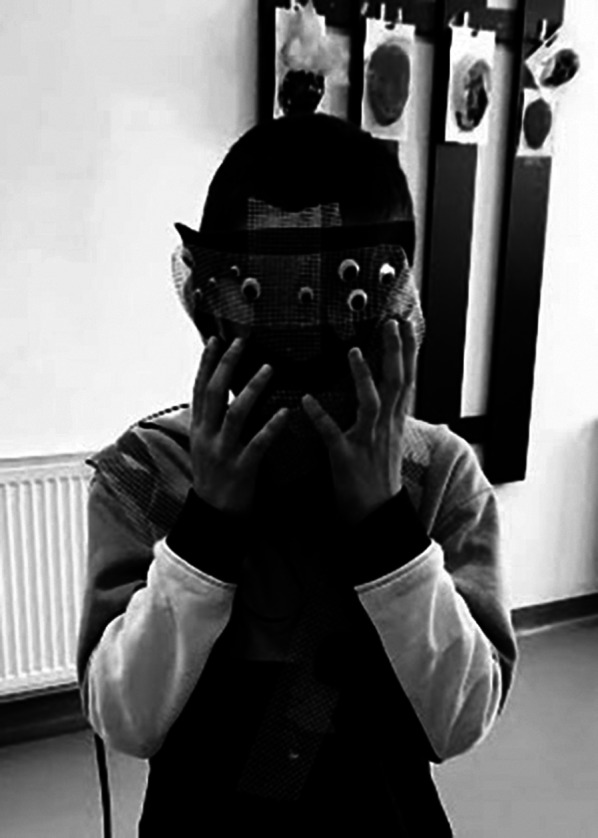

**Image 2:**

**Figure:**
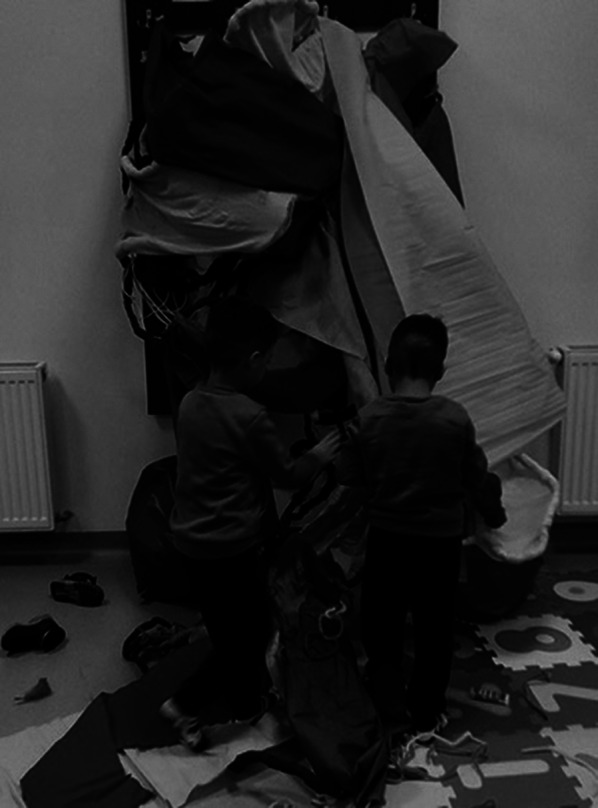

**Image 3:**

**Figure:**
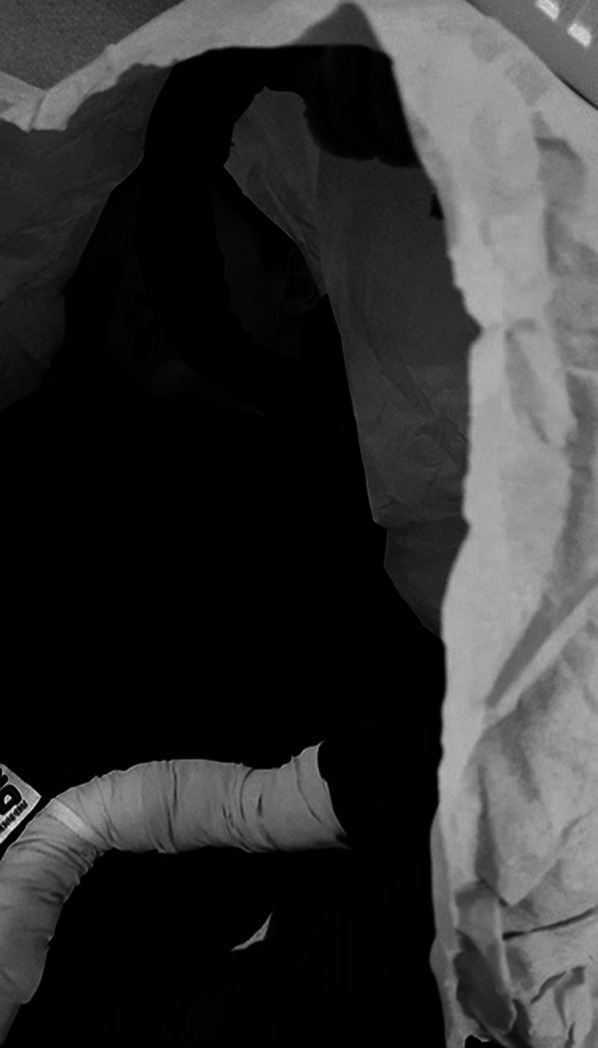

**Conclusions:**

The role of the artist/Art-therapist can be to identify and encourage the creative potential of the beneficiaries by making both individual and team artworks; to support social integration through art and to value the outstanding skills of the beneficiaries.

**Disclosure of Interest:**

None Declared

